# Early administration of glucocorticoid for thyroid storm: analysis of a national administrative database

**DOI:** 10.1186/s13054-020-03188-8

**Published:** 2020-07-29

**Authors:** Atsushi Senda, Akira Endo, Hisateru Tachimori, Kiyohide Fushimi, Yasuhiro Otomo

**Affiliations:** 1 Department of Acute Critical Care and Disaster Medicine, Graduate School of Medical and Dental Sciences, Tokyo Medical and Dental University, 1-5-45 Yushima, Bunkyo-ku, Tokyo, 113-8510 Japan; 2 Department of Mental Health Policy and Evaluation, National Institute of Mental Health, National Center of Neurology and Psychiatry, 4-1-1 Ogawahigashi, Kodaira, Tokyo, 187-0031 Japan; 3 Department of Health Policy and Informatics, Graduate School of Medical and Dental Sciences, Tokyo Medical and Dental University, 1-5-45 Yushima, Bunkyo-ku, Tokyo, 113-8510 Japan

**Keywords:** Thyroid storm, Glucocorticoid, Insulin use, Case-mix classification model, Bayesian statistics, Propensity score matching

## Abstract

**Background:**

Thyroid storm is a life-threatening disease with a mortality rate of over 10%. Although glucocorticoids have been recommended as a treatment option for thyroid storm, supportive evidence based on a large-scale clinical research is lacking. The objective of the current study was to evaluate the beneficial effects of glucocorticoids in the treatment of patients with severe thyroid storm.

**Methods:**

A retrospective nationwide cohort study was conducted using a Japanese national administrative claims database. Patients admitted to intensive care units due to severe thyroid storm between the financial years 2013 and 2017 were included in the study. The primary outcome was in-hospital mortality; secondary outcomes were mortality within 30 days and insulin administration during hospitalization. Generalized linear mixed model (GLMM) with maximum likelihood estimation (MLE) and Bayesian estimation using Markov chain Monte Carlo methods (MCMC), in addition to propensity score matching (PSM), were used for statistical analysis.

**Results:**

A total of 811 patients were included in the study, of which 600 patients were treated with glucocorticoids, and 211 patients were treated without glucocorticoids. The early administration of glucocorticoids was not associated with a significant improvement in the in-hospital mortality of patients with thyroid storm [adjusted odds ratio (95% confidence interval) = 1.77 (0.95–3.34), 1.44 (1.14–1.93), and 1.46 (0.72–3.00) in the GLMM (MLE), GLMM (MCMC), and PSM, respectively]. The results of mortality within 30 days were almost identical to the results of in-hospital mortality. However, insulin use was significantly higher in the glucocorticoid group.

**Conclusions:**

This analysis of a nationwide administrative database indicates that the administration of glucocorticoids does not improve the survival of patients with thyroid storm.

## Background

Thyroid storm (TS), also referred to as a thyroid crisis, is a life-threatening syndrome occurring due to the exacerbation of thyrotoxicosis. It is often triggered by severe stress in patients with thyrotoxicosis and is diagnosed by the presence of severe and life-threatening symptoms such as loss of consciousness, high fever, heart failure, diarrhea, and jaundice. TS causes disseminated intravascular coagulation, multiple organ failure, and shock, which lead to death in over 10% of the patients [1–5].

Treatment for TS mainly comprises agents that suppress excessive thyroid hormones, including anti-thyroid drugs (ATDs) and iodine. Further, some therapeutic options, such as beta-blockers, glucocorticoids, bile acid sequestrants, and plasmapheresis, are being used in a clinical setting for the treatment of TS. However, the evidence for all the abovementioned treatment options is based only on clinical experience or case studies analyzing a limited number of patients [6]. Glucocorticoids have been used as one of the treatment options for TS as they reduce the conversion of thyroxine (T4) to active triiodothyronine (T3). The typically recommended doses of glucocorticoids in this condition are hydrocortisone 100 mg every 6–8 h (300–400 mg/day) or dexamethasone 2 mg IV every 6 h (8 mg/day) continued until the resolution of the TS [7]. It was also shown that thyrotoxicosis is associated with subtle impairment of adrenocortical reserve [8]; therefore, glucocorticoids may benefit in this situation. However, glucocorticoids have also been reported to have potential risk for unfavorable effects such as hyperglycemia and immunodeficiency, which can worsen patient outcomes [9].

Owing to the low prevalence of the disease, limited clinical studies have been conducted on the treatments of TS. This study aimed to evaluate the clinical benefit of glucocorticoid treatment in patients with severe TS using a large-scale national administrative database.

## Materials and methods

### Study design, settings, and data sources

We conducted a retrospective cohort study to evaluate the efficacy of glucocorticoids for the treatment of patients with TS, using the Japanese Diagnosis Procedure Combination (DPC) database. The database is a case-mix classification system developed to maintain a prospective payment system for acute-phase inpatient hospital care, in which more than 1800 hospitals participated in the year 2014. The database contains the following information: hospital identification codes, patient demographics including sex, age, body weight, and date of admission, the score of activity of daily living, admission and discharge status, and post-admission complications, as well as main and secondary diagnoses. It also contains claims for every drug administered, every procedure, and care provided for each patient during hospitalization on a daily basis. Diagnoses were independently recorded using the relevant codes from the International Classification of Diseases, 10th revision (ICD-10). More details regarding the DPC database have been described elsewhere [10, 11]. The present study was conducted in accordance with the principles of the 1964 Declaration of Helsinki and its later amendments. The institutional review board of the Tokyo Medical and Dental University approved this study (#788). The requirement for informed consent from each patient was waived because of the retrospective design of the study, the use of anonymized patient, and hospital data.

### Study population

Patients with TS who were admitted to a DPC participating hospital between April 1, 2013, and March 31, 2017, were included in the study. The following participants were excluded from the study to homogenize the treatment quality: (i) patients younger than 16 years of age, (ii) patients with missing values in any variables used in the analyses, and (iii) patients not admitted to a government-approved specialized intensive care unit (ICU). In addition, patients who were discharged within 3 days of admission were also excluded to avoid immortal-time bias as the patient severity was adjusted by the intensity of treatments provided within 3 days of admission.

### Data collection

The following data were collected from the DPC database: age; sex; ICD-10 codes for four primary diagnoses, concurrent diagnoses at admission, and post-admission complications; unique hospital identifier; the annual number of patients with TS per hospital; status at discharge from hospital (i.e., survived or deceased); and duration of hospitalization. We collected information on whether the following interventions were performed within 3 days of admission: mechanical ventilation, renal replacement therapy, intra-aortic balloon pumping, extracorporeal membrane oxygenation (ECMO), cardioversion, and plasmapheresis. Information on the administration of glucocorticoids (hydrocortisone, dexamethasone, methylprednisolone, prednisolone, or betamethasone), vasopressors (dopamine, norepinephrine, epinephrine, or vasopressin), acetaminophen, methimazole, potassium iodide, and beta-blockers were also collected. The dose of vasopressors to be used was estimated based on Vasoactive-Inotropic Score (VIS) [12], which was originally developed for pediatric patients but has been validated for use in adults and has been used in several studies [13–15]. The comorbidities of patients were assessed using the Charlson comorbidity index [16] based on previously reported methods for extracting the ICD-10 codes [17]. The dose of each glucocorticoid was converted to hydrocortisone equivalent [18, 19].

### Definitions and outcomes

The glucocorticoid treatment group included the patients who were administered any kind of glucocorticoid within 3 days of admission regardless of amount. The primary outcome was in-hospital mortality. Secondary outcomes were mortality within 30 days and insulin use after admission. We chose insulin use as the surrogate outcome of glucose tolerance impairment because blood glucose values were not available in the DPC database.

### Statistical analysis

The machine-learning algorithm XGBoost [20] was used to estimate the importance of glucocorticoid use on the primary outcome. The algorithm was defined by the following parameters: base score, 0.5; booster, gbtree; node, 1; importance type, gain; learning rate, 0.001; maximum depth, 3; and numbers of estimators, 100.

Considering the heterogeneity of disease severity, we developed a risk adjustment model for in-hospital mortality using the variables, age, sex, Charlson comorbidity index, and level of consciousness (alert or not). We also included the following interventions performed within 3 days of admission to estimate the treatment intensity: renal replacement therapy, mechanical ventilation, defibrillation, ECMO, VIS calculated by the dose of administered vasopressors, the use of acetaminophen, beta-blockers, iodine, and ATDs, by applying a logistic regression model that included a random sample of 80% of the entire study cohort. The covariables were selected based on the clinical perspective from previous studies [5, 21] and the ten events per variable rule. Issues with variable multicollinearity were assessed by a variance inflation factor (VIF), and the tolerance value was set at less than 2. The prognostic accuracy of the model was validated in the remaining 20% of the cohort using the area under the receiver operating characteristics curve (AUROC) and Hosmer-Lemeshow goodness-of-fit test.

The efficacy of glucocorticoid treatment was assessed using three models: a mixed-effects logistic regression model [22], a mixed-effects model using the Bayesian method, the Markov chain Monte Carlo (MCMC) method [23], and propensity score matching analysis [24]. For the mixed-effects logistic regression model, the established case-mix classification model was used to adjust differences in disease severity in each patient, while simultaneously controlling hospital-level clustering. For the Bayesian model, no prior information was used; however, other hyperparameters used were iteration: 2000, burn in: 1000, and chains: 4. Although these hyperparameters were the default values in the software, their validity was confirmed by fulfilling the convergence judgment: Rhat < 1.1 [25]. Additionally, the consistency of the posterior distribution between each chain was confirmed. The Bayes factor was calculated from the posterior distribution [26]. The propensity score for predicting glucocorticoid use was calculated by logistic regression analysis using the variables used for the establishment of aforementioned prognosis model, annual number of TS cases per hospital, whether it was an academic hospital, and number of ICU beds to account for the differences in treatment quality at each hospital. Propensity score matching extracted 1:1 matched pairs from the glucocorticoid group and the control group. Match balance between the two groups was assessed using the absolute standardized mean difference (ASMD), which is calculated as the absolute value in the difference in the means of a covariate across the two groups divided by the standard deviation in the treated group. Of all variables, values lower than 0.1 were regarded as acceptable [27, 28]. To achieve the balanced matching, the caliper width for matching was set as the standard deviation (SD) of the logit-transformed propensity score multiplied by 0.1. Intergroup comparisons of the outcomes with propensity score-matched subjects were performed using the chi-squared test.

The sensitivity analysis assessed the patients who received glucocorticoids for the purpose of treatment of catecholamine-resistant septic shock. We compared the primary outcomes (i.e., in-hospital mortality) between patients who received > 200 mg/day of hydrocortisone and patients who did not receive any glucocorticoid or received ≤ 200 mg/day of hydrocortisone (≤ 200 mg/day group). The cut-off value was determined based on the dose of glucocorticoid recommended by the surviving sepsis campaign guideline [29].

Statistical analyses were performed using R software (version 3.6.0; R Foundation for Statistical Computing, Vienna, Austria). Bayesian regression was performed using package RStan (version 2.19.2). XGBoost was performed using Python (version 3.7.3). Intergroup comparison of the outcomes was performed using a Fisher’s exact test. The level of significance was defined by a two-sided value of *p* < 0.05. A Bayesian confidence interval was used for estimating the 95% confidence interval in the Bayesian analysis.

## Results

**Fig. 1:**
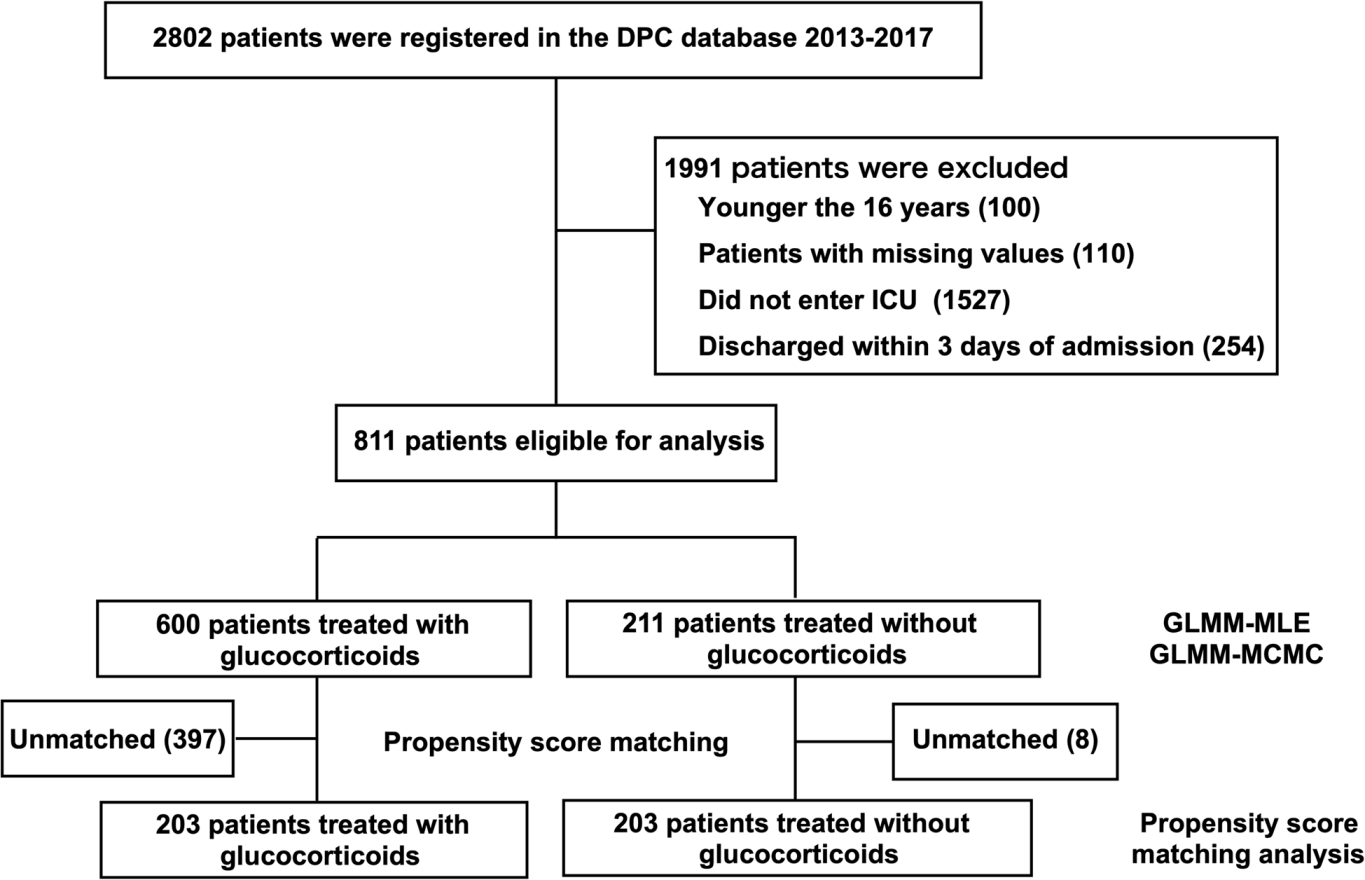
Patient flow diagram. Abbreviations: DPC database, Japanese Diagnosis Procedure Combination database; ICU, intensive care unit; GLMM, generalized linear mixed model; MLE, maximum likelihood estimation; MCMC, Markov chain Monte Carlo

The flow diagram of the patient selection process is shown in Fig. 1. During the study period, 2802 patients with TS were hospitalized at DPC participating hospitals. Among them, 811 patients were included in the study based on inclusion and exclusion criteria. Among 811 patients, 600 received glucocorticoid treatment within 3 days of admission. Patient characteristics based on the administration of glucocorticoid treatment are shown in Table 1. The overall mortality rate was 13.4% (109/811), and overall mortality within 30 days was 11.1% (90/811). The in-hospital mortality rate was 15.2% (91/600) in the glucocorticoid treatment group and 8.5% (18/211) in the control group (*p* = 0.014). The 30-day mortality rate was 12.3% (74/600) in the glucocorticoid treatment group, and 7.6% (16/211) in the control group (*p* = 0.073). The rate of patients who received insulin was 27.5% (165/600) in the glucocorticoid treatment group and 25.1% (53/211) in the control group (*p* = 0.041).

**Table 1:** Characteristics of patients and hospitals

Patient or hospital characteristic	Glucocorticoid group	Control group
Number of participants, *n*	600	211
Age (years), median [25th–75th percentiles]	49 [38, 60]	51 [35, 67]
Sex, female, *n* (%)	429 (71.5)	127 (60.2)
Charlson comorbidity index, median [25th–75th percentiles]	1 [0, 1]	1 [0, 2]
Vasoactive-Inotropic Score, median [25th–75th percentiles]	0 [0, 0]	0 [0, 0]
Consciousness, alert, *n* (%)	338 (56.3)	119 (56.4)
Defibrillation, *n* (%)	33 (5.5)	6 (2.8)
Mechanical ventilation use, *n* (%)	208 (34.7)	54 (25.6)
Renal replacement therapy, *n* (%)	58 (9.7)	4 (1.9)
Plasma exchange, *n* (%)	24 (4.0)	0 (0.0)
Extra-corporeal membrane oxygenation *n* (%)	27 (4.5)	3 (1.4)
Intra-aortic balloon pump, *n* (%)	21 (3.5)	4 (1.9)
Acetaminophen use, *n* (%)	213 (35.5)	54 (25.6)
Beta-blockers use, *n* (%)	421 (70.1)	112 (53.1)
Methimazole use, *n* (%)	512 (85.3)	122 (57.8)
Potassium Iodide use, *n* (%)	432 (72.0)	84 (39.8)
Annual number of patients of thyroid storm per hospital, median [25th–75th percentiles]	7 [4, 10]	7 [3, 10]
Admission number of ICU each day per hospital, median [25th–75th percentiles]	5.4 [3.1, 7.4]	5.1 [3.0, 8.3]
Academic hospital, *n* (%)	137 (22.8)	50 (23.7)

*Abbreviations*: *ASMD* absolute standard mean difference, *ICU* intensive care unit

The proportion of patients who received renal replacement therapy and mechanical ventilation was higher in the glucocorticoid treatment group compared to the control group. The result of feature importance by XGBoost is shown in Supplemental Figure 1. Together with age, VIS, and Charlson comorbidity index, glucocorticoid use showed a high feature importance value, which suggests that glucocorticoid use was an important factor for predicting patient survival at the time of discharge. The VIFs of all variables used in the regression analysis were less than 2, which eliminated the issue of multicollinearity in our model. The established case-mix classification model was well calibrated for the validation cohort (AUROC 0.84; Hosmer-Lemeshow goodness-of-fit test *p* = 0.873) (Supplemental Figure 2).

**Fig. 2:**
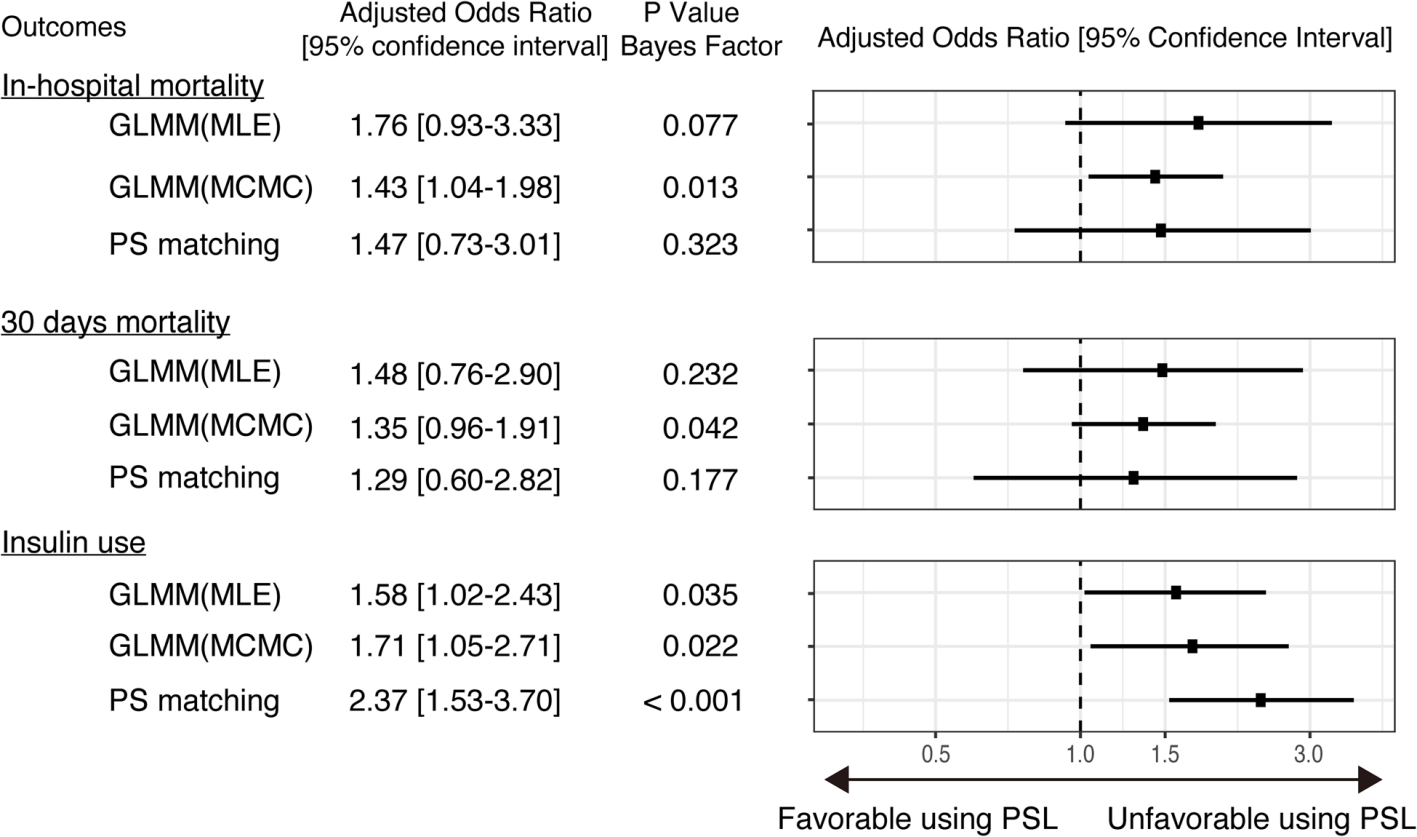
Forest plot of each analysis comparing patients treated with and without glucocorticoids. Abbreviations: GLMM, generalized linear mixed model; MLE, maximum likelihood estimation; MCMC, Markov chain Monte Carlo; PS, propensity score

Results of analyses by three models evaluating the efficiency of glucocorticoid use are summarized in Fig. 2. The mixed-effects logistic regression analysis demonstrated that the difference was not statistically significant for in-hospital mortality [adjusted odds ratio (95% confidence interval; CI) = 1.77 (0.95–3.34)] or for the 30-day mortality rate [adjusted odds ratio (95% CI) = 1.48 (0.75–2.92)]. A significantly higher frequency of insulin use was observed in the glucocorticoid treated group [adjusted odds ratio (95% CI) = 1.56 (1.00–2.43)]. Bayesian analysis revealed similar results as those found with the mixed-effects regression model. Increased mortality was observed in the glucocorticoid group as demonstrated by in-hospital mortality [adjusted odds ratio (Bayesian 95% CI) = 1.44 (1.14–1.93)] and mortality within 30 days [adjusted odds ratio (Bayesian 95% CI) = 1.34 (0.95–1.91)]. A higher frequency of insulin use was observed in the glucocorticoid treated group [adjusted odds ratio (Bayesian 95% CI) = 1.71 (1.06–2.77)]. The density distribution of regression coefficients of all covariates is listed in Supplemental Figure 3.

The patient characteristics of propensity score-matched samples are shown in Table 2. Overall, 203 patients were matched based on the propensity score. Among the matched groups, ASMD < 0.1 was observed in all the covariates, indicating that the baseline characteristics of the glucocorticoid treatment and control groups were sufficiently similar. The in-hospital mortality rate was 8.4% (17/203) in the glucocorticoid treatment group and 11.8% (24/203) in the control group. The 30-day mortality rate was 7.4% (15/203) in the glucocorticoid treatment group and 9.4% (19/203) in the control group. The rate of insulin use was 44.3% (90/203) in the glucocorticoid treatment group and 25.1% (51/203) in the control group.

**Table 2:** Patient characteristics of patients after propensity score matching

	Glucocorticoid group	Control group	ASMD
Number of participants, *n*	203	203	
Age(years), median [25th–75th percentiles]	52 [41, 64]	50 [35, 66]	0.08
Sex, female, *n* (%)	131 (64.5)	127 (62.6)	0.04
Charlson comorbidity index, median [25th–75th percentiles]	1 [0, 1]	1 [0, 1]	< 0.01
Vasoactive-Inotropic Score (VIS), median [25th–75th percentiles]	0 [0, 0]	0 [0, 0]	0.08
Consciousness, alert, *n* (%)	57 (50.0)	56 (50.0)	0.03
Defibrillation, *n* (%)	6 (3.0)	6 (3.0)	< 0.01
Mechanical ventilation use, *n* (%)	57 (28.1)	52 (25.6)	0.06
Renal replacement therapy, *n* (%)	6 (3.0)	4 (2.0)	0.06
Extra-corporeal membrane oxygenation, *n* (%)	4 (2.0)	3 (1.5)	0.05
Intra-aortic balloon pump, *n* (%)	4 (2.0)	4 (2.0)	< 0.01
Acetaminophen use, *n* (%)	58 (28.5)	50 (24.6)	0.04
Beta-blockers use, *n* (%)	108 (53.2)	107 (52.7)	< 0.01
Methimazole use, *n* (%)	111 (54.7)	112 (55.2)	< 0.01
Potassium Iodide use, *n* (%)	83 (40.9)	83 (40.9)	0.01
Annual number of patients with thyroid storm per hospital, median [25th–75th percentiles]	7 [4, 10]	7 [3, 10]	0.06
Admission number of ICU each day per hospital, median [25th–75th percentiles]	5.4 [3.3, 7.8]	5.0 [2.9, 7.8]	0.01
Academic hospital, *n* (%)	46 (22.7)	45 (22.2)	0.01

*Abbreviation*: *ASMD* absolute standard mean difference, *ICU* intensive care unit

A statistically significant difference was not observed in both in-hospital and 30-day mortality rates. Insulin administration was significantly more frequent in the glucocorticoid treated group [adjusted odds ratio (95% CI) = 2.39 (1.54–3.72)] (Fig. 2).

**Fig. 3:**
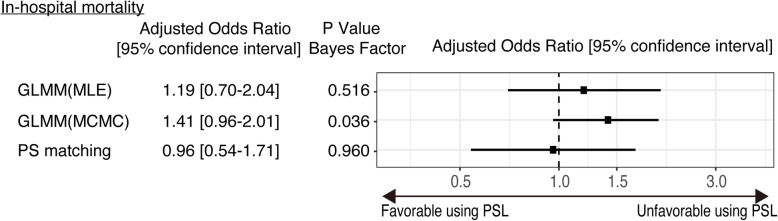
Forest plot of each analysis comparing patients treated with low-dose glucocorticoid and high-dose glucocorticoid. Abbreviations: GLMM, generalized linear mixed model; MLE, maximum likelihood estimation; MCMC, Markov chain Monte Carlo; PS, propensity score

The results of the sensitivity analysis that compared in-hospital mortality between the > 200 mg/day group [569 patients (70.2%)] and ≤ 200 mg/day group [242 patients (29.8%)] are summarized in Fig. 3. Statistically significant differences were not observed between the > 200 mg/day group and the ≤ 200 mg/day group [adjusted odds ratio (95% CI) = 1.21 (0.74–2.03), 1.41 (0.96–2.00), and 0.96 (0.54–1.72) in GLMM (MLE), GLMM (MCMC), and propensity score matching, respectively].

## Discussion

Owing to the low prevalence of TS, studies on causal inference are scarce in this field, and treatments have been performed based on several descriptive research studies [1, 5, 21]. To the best of our knowledge, the present study is the first to evaluate the efficacy of TS treatment by analyzing a large-scale nationwide database.

Glucocorticoids are considered to be provided with a “strong recommendation with low-quality evidence” in American guidelines, and a “high strength of recommendation with moderate evidence” in Japanese guidelines [30, 31]. These recommendations have been validated from the improved outcomes in a case series of 22 patients with TS [32]. Glucocorticoids reduce the conversion of T4 to active T3 and may simultaneously improve the underlying autoimmune process [8]. However, the Japanese guidelines state that the overuse of glucocorticoids may cause unfavorable hyperglycemia and immunosuppressive conditions. The results in the current study did not show a statistically significant difference in survival outcomes by glucocorticoid use. Instead, a significant association between early glucocorticoid use and insulin use was seen, which might be related to glucose tolerance abnormalities.

The baseline characteristics of TS patients were almost identical to the previously reported Japanese nationwide multicenter observational study [1]. However, the mortality rate observed in this study was higher as compared to the previous study (13.5% vs. 10.1%). This difference might be explained by the target population. The patients who did not enter a government-approved specialized ICU were excluded from this study to homogenize the quality of care provided. In addition to the mortality rate, this study provides detailed treatment approaches performed in current Japanese settings. Taking into consideration the devastating clinical course of TS, there is a need to accumulate evidence for treatment strategies of TS. Although the current study was a retrospective analysis and patient severity differed between the two groups, we used several statistical models. As it is impractical to perform a randomized controlled trial or a large-scale prospective study due to the rarity of the disease, the present study that analyzed large-scale real-world data, would be noteworthy in this field and can add evidence pertaining to the treatment strategy of TS. We used the Bayesian statistics for data analyses, and the data of posterior probability distribution in the present study can be used as a prior distribution in future research works in this field. Furthermore, considering the heterogeneity between the two groups, a propensity score matching analysis was also performed. Approximately 400 patients in the glucocorticoid treatment group were excluded by the matching process to achieve a well-matched balance, the majority of which were suggested as severe cases from the characteristics of the propensity score-matched cohort. However, the statistical models other than the propensity score matching model analyzed the entire study cohort and adjusted patient severity using the well-calibrated case-mix adjustment model, such that the study results were robust.

TS is frequently triggered by severe stress, such as septic shock, in patients with thyrotoxicosis. Glucocorticoids have been used in patients with catecholamine-resistant septic shock [33, 34]. According to the guidelines of the surviving sepsis campaign [29], the recommended dose of glucocorticoids in such patients is 200 mg/day of hydrocortisone, whereas 300–400 mg/day of hydrocortisone is typically recommended in TS [7]. Given the possibility of underestimation of the glucocorticoid treatment for TS due to patients with catecholamine-resistant septic shock, we performed a sensitivity analysis to compare ≤ 200 mg/day group with the > 200 mg/day group; however, no apparent prognostic disadvantage was noted in the ≤ 200 mg/day group even though their prognoses were poor. Although this result suggested that the population with poor prognosis (i.e., TS patients complicated by catecholamine-resistant septic shock) did not skew the results of the main analyses, those patients might have benefited from low-dose glucocorticoids. Although most glucocorticoid-treated patients were treated with hydrocortisone (85.3%), the remaining 14.7% of patients were treated with other glucocorticoids: these were converted to the equivalent hydrocortisone dose in this analysis. The optimal conversion ratio is still debatable, and the difference in the glucocorticoid effect depending on the dose could not be assessed in the present study. Further study is required to assess the effect of glucocorticoids in patients with TS, considering the detailed information of the dose.

Plasmapheresis has also been used as a treatment strategy for severe TS cases; however, supportive evidence for this and other treatment options is scarce. Plasmapheresis involves the removal and exchange of serum proteins to which 99% of the thyroid hormones bind [35]. In the present study, although we analyzed a large-scale database, the number of patients who underwent plasmapheresis was only 42, which was insufficient to evaluate its effectiveness using multivariate analysis. Further large-scale studies would be required to evaluate the efficacy of other treatment options, including plasmapheresis, for severe TS patients.

Our study has several limitations. First, the recorded diagnoses in the database are less well validated than in planned prospective studies. Although there is a study showing that the specificity of the diagnosis in the DPC database exceeded 96% [36], we could not confirm whether the patients fulfilled the Japan Thyroid Association criteria or Burch-Wartofsky Point Scale in the diagnosis of thyroid storm [1, 37]. However, our data of patient characteristics and administered treatments were similar to the study analyzing patients that fulfilled the diagnostic criteria [1], indicating that the analyzed population could reflect patients with TS to a considerable extent. Second, we excluded patients discharged within 3 days, who may have been unable to receive enough intensity of care including glucocorticoid therapy, although the consequence of this bias was uncertain. Third, although we used the well-validated predictive model in the analysis, the possibility of residual confounding factors existed because of the retrospective nature of the study and the used database in which vital signs and results of laboratory tests were not available. Fourth, as this is the first study estimating the efficacy of glucocorticoids in TS treatment, prior sample size estimation was impossible. Fifth, although we showed the significant association between early glucocorticoid use and insulin use, insulin use was just a surrogate outcome, and it did not directly mean the impairment of glucose tolerance. Finally, details of the underlying condition for TS (e.g., sepsis), which might have affected the patient outcomes, were not always available in the DPC database. However, to the best of our knowledge, this is the first well-designed large-scale study on patients with TS using the case-mix classification model with high accuracy for predicting mortality. Our results suggest that the routine use of glucocorticoids does not improve the survival of patients with TS, indicating that clinicians should consider glucocorticoid use on an individual patient basis, accounting for the risks of infection and hyperglycemia. Larger retrospective and prospective studies are necessary to support our findings and to determine which subgroups of patients could benefit from glucocorticoids.

## Conclusions

Our analysis of a nationwide administrative database indicates that the administration of glucocorticoids does not improve the survival of patients with TS. Insulin was more frequently used in patients who received glucocorticoids. Further evidence of glucocorticoid use might be desirable for TS patients.

## Supplementary information

**Additional file 1 : Supplemental Figure 1**. Feature importance measured by XGBoost.

**Additional file 2 : Supplemental Figure 2**. Goodness of fit of case-mix classification model. (A) Receiver operating curves of risk adjustment model in the validation cohort. AUROC area under the receiver operating curve. (B) Hosmer-Lemeshow goodness-of-fit test.

**Additional file 3 : Supplemental Figure 3**. Posterior density distribution of each coefficient of regression model (A) Regression coefficient at the level of patient, (B) Regression coefficient at the level of hospitals.

## Data Availability

The datasets used and/or analyzed during the current study are available from the corresponding author on reasonable request.

## Abbreviations

ASMD: Absolute standardized mean difference
ATDs: Anti-thyroid drugs
AUROC: Area under the receiver operating characteristics
DPC Database: Diagnosis Procedure Combination database
ECMO: Extracorporeal membrane oxygenation
GLMM: Generalized linear mixed model
ICD-10: International Classification of Diseases, 10th revision
ICU: Intensive care unit
MCMC: Markov chain Monte Carlo
MLE: Maximum likelihood estimation
PSM: Propensity score matching
SD: Standard deviation
TS: Thyroid storm
T3: Triiodothyronine
T4: Thyroxine
VIF: Variance inflation factor
VIS: Vasoactive-Inotropic Score

## References

[1] Akamizu T, Satoh T, Isozaki O, Suzuki A, Wakino S, Iburi T (2012). Diagnostic criteria, clinical features, and incidence of thyroid storm based on nationwide surveys. Thyroid..

[2] Isozaki O, Satoh T, Wakino S, Suzuki A, Iburi T, Tsuboi K (2016). Treatment and management of thyroid storm: analysis of the nationwide surveys: the taskforce committee of the Japan Thyroid Association and Japan Endocrine Society for the establishment of diagnostic criteria and nationwide surveys for thyroid storm. Clin Endocrinol.

[3] Swee du S, Chng CL, Lim A (2014). Clinical characteristics and outcome of thyroid storm: a case series and review of neuropsychiatric derangements in thyrotoxicosis. Endocr Pract.

[4] Angell TE, Lechner MG, Nguyen CT, Salvato VL, Nicoloff JT, LoPresti JS (2015). Clinical features and hospital outcomes in thyroid storm: a retrospective cohort study. J Clin Endocrinol Metab.

[5] Ono Y, Ono S, Yasunaga H, Matsui H, Fushimi K, Tanaka Y (2016). Factors associated with mortality of thyroid storm: analysis using a national inpatient database in Japan. Med.

[6] Chiha M, Samarasinghe S, Kabaker AS (2015). Thyroid storm: an updated review. J Intensive Care Med.

[7] Carroll R, Matfin G (2010). Endocrine and metabolic emergencies: thyroid storm. Ther Adv Endocrinol Metab.

[8] Tsatsoulis A, Johnson EO, Kalogera CH, Seferiadis K, Tsolas O (2000). The effect of thyrotoxicosis on adrenocortical reserve. Eur J Endocrinol.

[9] Stewart PM, JDC N.-P. The adrenal cortex. In: Melmed S, Polonsky K. S, Larsen P. R, Kronenberg H. M, editors. Williams textbook of endocrinology.13. Philadelphia: Elsevier; 2016. p.490–555.

[10] Yasunaga H, Matsui H, Horiguchi H, Fushimi K, Matsuda S (2015). Clinical epidemiology and health services research using the diagnosis procedure combination database in Japan. Asian Pac J Dis Manag.

[11] Ishikawa KB (2016). Medical big data for research use: current status and related issues. Jpn Med Assoc J.

[12] Gaies MG, Jeffries HE, Niebler RA, Pasquali SK, Donohue JE, Yu S (2014). Vasoactive-inotropic score is associated with outcome after infant cardiac surgery: an analysis from the Pediatric Cardiac Critical Care Consortium and Virtual PICU System Registries. Pediatr Crit Care Med.

[13] Yamazaki Y, Oba K, Matsui Y, Morimoto Y (2018). Vasoactive-inotropic score as a predictor of morbidity and mortality in adults after cardiac surgery with cardiopulmonary bypass. J Anesth.

[14] Lim JY, Kim JB, Jung SH, Choo SJ, Chung CH, Lee JW (2017). Risk factor analysis for nonocclusive mesenteric ischemia following cardiac surgery: a case-control study. Med.

[15] Nguyen HV, Havalad V, Aponte-Patel L, Murata AY, Wang DY, Rusanov A (2013). Temporary biventricular pacing decreases the vasoactive-inotropic score after cardiac surgery: a substudy of a randomized clinical trial. J Thorac Cardiovasc Surg.

[16] Charlson ME, Pompei P, Ales KL, MacKenzie CR (1987). A new method of classifying prognostic comorbidity in longitudinal studies: development and validation. J Chronic Dis.

[17] Quan H, Sundararajan V, Halfon P, Fong A, Burnand B, Luthi JC (2017). Coding algorithms for defining comorbidities in ICD-9-CM and ICD-10 administrative data. Med Care.

[18] Webb R, Singer M (2005). Oxford Handbook of Critical Care.

[19] Meikle AW, Tyler FH (1977). Potency and duration of action of glucocorticoids. Am J Med.

[20] Tianqi Chen, Carlos Guestrin. XGBoost: A Scalable Tree Boosting System. Proceedings of the 22nd ACM SIGKDD international conference on knowledge discovery and data mining, San Francisco, CA, USA, August 13–17. pp 785–794.

[21] Galindo RJ, Hurtado CR, Pasquel FJ, Garcia Tome R, Peng L (2019). National trends in incidence, mortality, and clinical outcomes of patients hospitalized for thyrotoxicosis with and without thyroid storm in the United States, 2004-2013. Thyroid..

[22] Liang KY, Zeger SL (1986). Longitudinal data analysis using generalized linear models. Biometrika.

[23] Fong Y, Rue H, Wakefield J (2010). Bayesian inference for generalized linear mixed models. Biostat..

[24] D’Agostino RB (1998). Propensity score methods for bias reduction in the comparison of a treatment to a non-randomized control group. Stat Med.

[25] Gelman A, Carlin JB, Stern HS, Rubin DB (2004). Bayesian data analysis.

[26] Goodman SN (1999). Toward evidence-based medical statistics. 2: the Bayes factor. Ann Intern Med.

[27] Stuart EA, Lee BK, Leacy FP (2013). Prognostic score–based balance measures can be useful diagnostic for propensity score methods in comparative effectiveness research. J Clin Epidemiol.

[28] Rubin DB (2001). Using propensity scores to help design observational studies: application to the tobacco litigation. Health Serv Outcome Res Meth.

[29] Dellinger RP, Levy MM, Rhodes A, Annane D, Gerlach H, Opal SM (2013). Surviving sepsis campaign: international guidelines for management of severe sepsis and septic shock: 2012. Crit Care Med.

[30] Satoh T, Isozaki O, Suzuki A, Wakino S, Iburi T, Tsuboi K (2016). Guidelines for the management of thyroid storm from The Japan Thyroid Association and Japan Endocrine Society. Endocr J.

[31] Ross DS, Burch HB, Cooper DS, Greenlee MC, Laurberg P, Maia AL (2016). American Thyroid Association guidelines for diagnosis and management of hyperthyroidism and other causes of thyrotoxicosis. Thyroid..

[32] Mazzaferri EL, Skillman TG (1969). Thyroid storm: a review of 22 episodes with special emphasis on the use of guanethidine. Arch Intern Med.

[33] Lamontagne F, Rochwerg B, Lytvyn L, Guyatt GH, Møller MH, Annane D (2018). Corticosteroid therapy for sepsis: a clinical practice guideline. BMJ..

[34] Rochwerg B, Oczkowski SJ, Siemieniuk RAC, Agoritsas T, Belley-Cote E, D’Aragon F (2018). Corticosteroids in sepsis: an updated systematic review and meta-analysis. Crit Care Med.

[35] Wartofsky L, Braverman LE, Cooper DS (2005). Thyrotoxic storm. Werner & Ingbar’s the thyroid: a fundamental and clinical text.

[36] Yamana H, Moriwaki M, Horiguchi H, Kodan M, Fushimi K, Yasunaga H (2017). Validity of diagnoses, procedures, and laboratory data in Japanese administrative data. J Epidemiol.

[37] Burch HB, Wartofsky L (1993). Life-threatening thyrotoxicosis. Thyroid storm. Endocrinol Metab Clin N Am.

