# Supplementary figures and images for: Early administration of glucocorticoid for thyroid storm: analysis of a national administrative database
Source: Crit Care. 2020 Jul 29;24:470. doi: 10.1186/s13054-020-03188-8 (PMC7391822; doi:10.1186/s13054-020-03188-8)

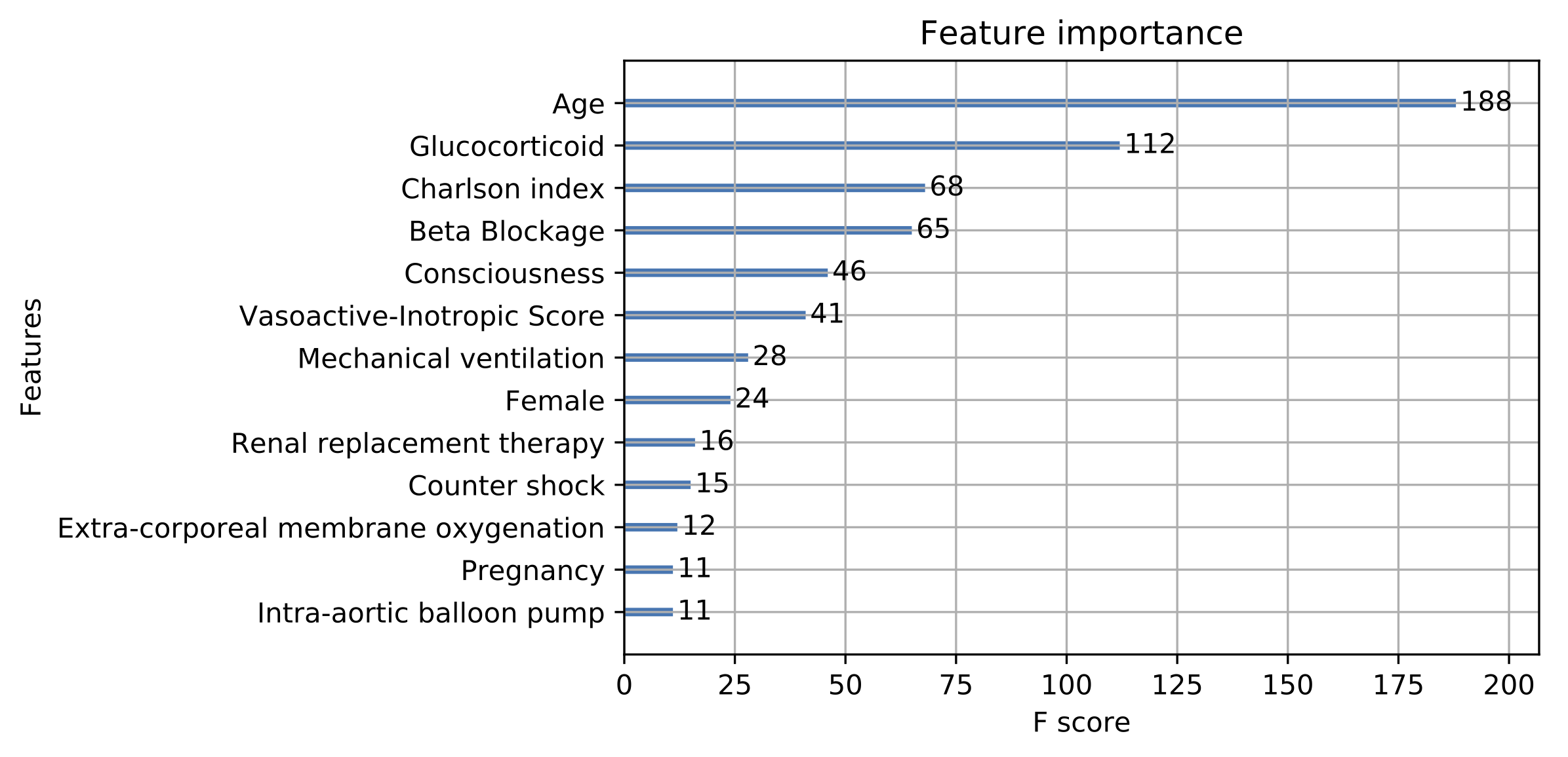

Supplement: Supplementary file 1 — Additional file 1 : Supplemental Figure 1. Feature importance measured by XGBoost. [file 13054_2020_3188_MOESM1_ESM.tiff]

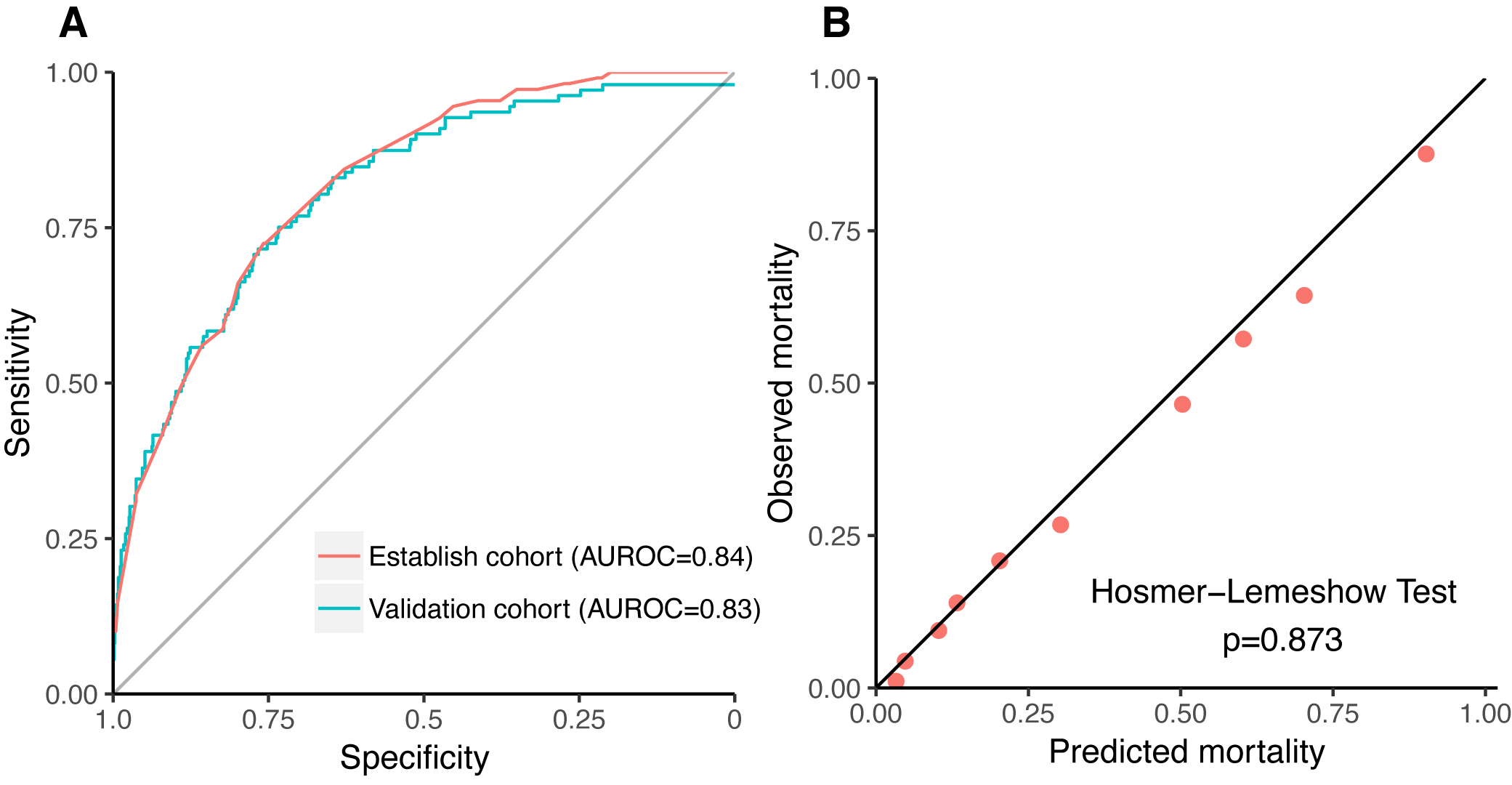

Supplement: Supplementary file 2 — Additional file 2 : Supplemental Figure 2. Goodness of fit of case-mix classification model. (A) Receiver operating curves of risk adjustment model in the validation cohort. AUROC area under the receiver operating curve. (B) Hosmer-Lemeshow goodness-of-fit test. [file 13054_2020_3188_MOESM2_ESM.tif]

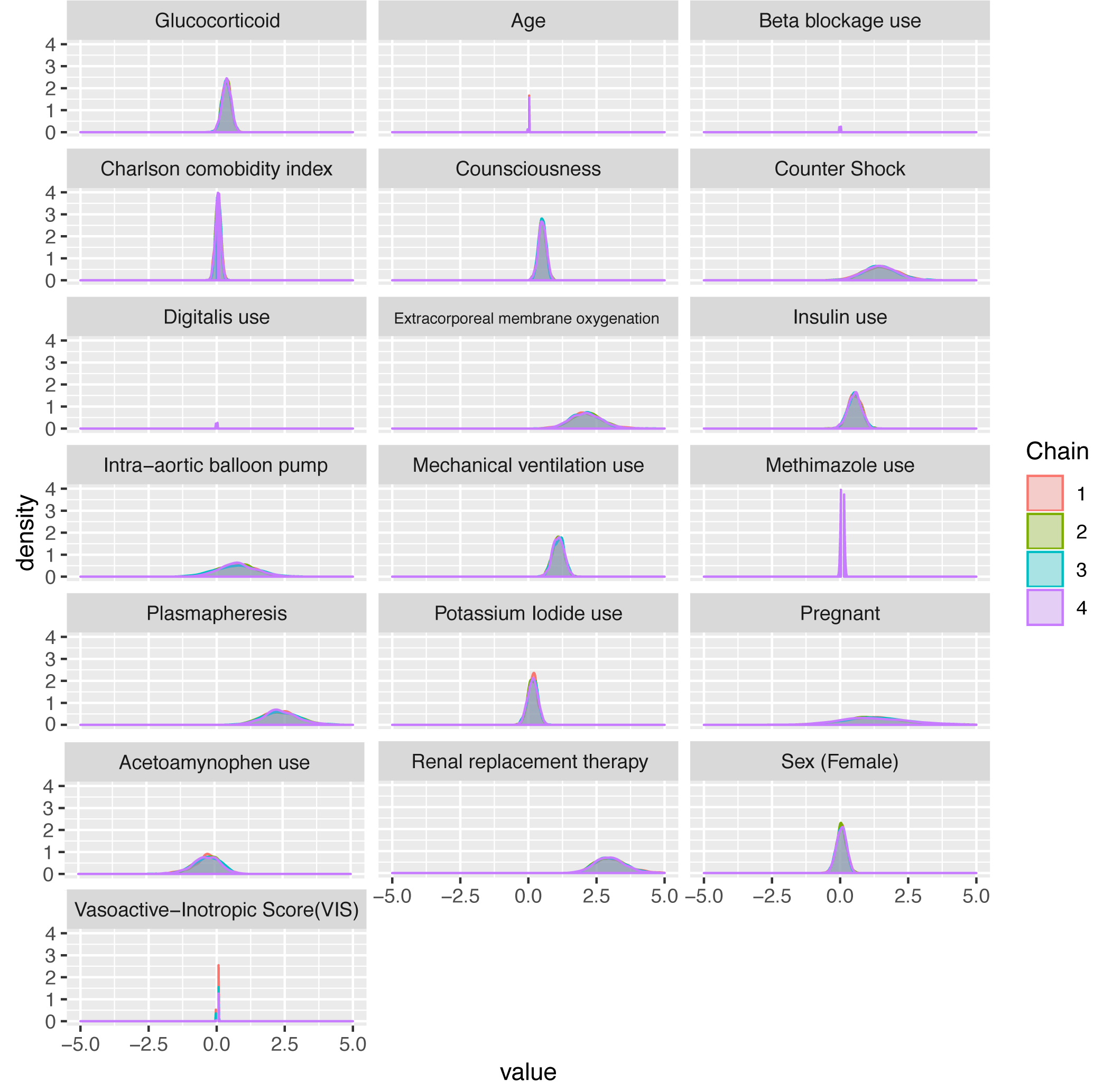

Supplement: Supplementary file 3 — Additional file 3 : Supplemental Figure 3. Posterior density distribution of each coefficient of regression model (A) Regression coefficient at the level of patient, (B) Regression coefficient at the level of hospitals. [file 13054_2020_3188_MOESM3_ESM.zip › Supplement3a.tif]

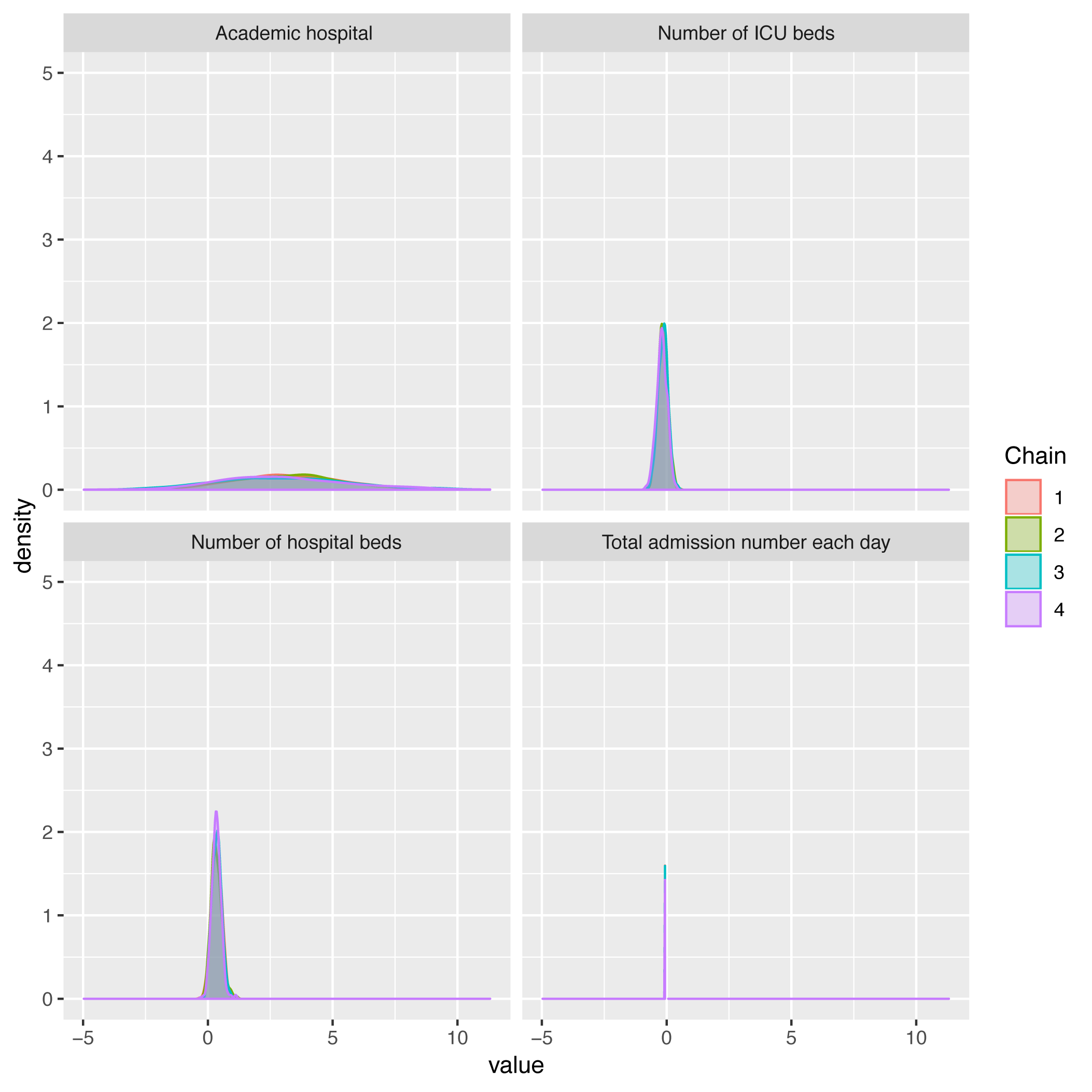

Supplement: Supplementary file 3 — Additional file 3 : Supplemental Figure 3. Posterior density distribution of each coefficient of regression model (A) Regression coefficient at the level of patient, (B) Regression coefficient at the level of hospitals. [file 13054_2020_3188_MOESM3_ESM.zip › Supplement3b.tif]
